# The interplay between sinus pathology and dental infections

**DOI:** 10.6026/973206300213475

**Published:** 2025-10-31

**Authors:** Ankur Bhargava, Timbadiya Vijaykumar Mansukhbhai, Nishad Gawali, Dakshayan Patil, Rashmi Laddha, Swati Kharat

**Affiliations:** 1 Department of Oral Pathology & Microbiology, Hazaribag College of Dental Sciences & Hospital, Hazaribagh, Jharkhand, India; 2 Department of Oral Pathology and Microbiology, Rishiraj College of Dental Sciences and Research Centre, Pipalner Road, Bhopal, Madhya Pradesh, India; 3 Department of Public Health Dentistry, Hazaribag College of Dental Sciences and Hospital, Hazaribagh, Jharkhand, India; 4 Department of Oral Pathology and Microbiology, Hazaribag College of Dental Sciences and Hospital, Hazaribagh, Jharkhand, India; 5 Department of Periodontics, Dr RR Kambe Dental College and Hospital, Shegaon Road, Akola, Maharashtra, India; 6 Department of Prosthodontics, Dr RR Kambe Dental College and Hospital, Shegaon Road, Akola, Maharashtra, India

**Keywords:** Odontogenic sinusitis, chronic rhinosinusitis, dental infection, maxillary sinus, cone-beam computed tomography, interdisciplinary management

## Abstract

The close anatomical relationship between the maxillary sinuses and posterior maxillary teeth fosters a bidirectional link between sinus pathology and a dental infection is of interest. Odontogenic maxillary sinusitis frequently arises from periapical disease, periodontal pathology, or dental procedures, while chronic rhinosinusitis can secondarily impact dental structures. Overlapping symptoms often obscure diagnosis, but advanced imaging such as cone-beam computed tomography enables accurate source identification. Early detection and targeted management of the primary pathology are vital to preventing chronicity and recurrence. Interdisciplinary collaboration between dental and otolaryngology specialists remains essential for achieving optimal outcomes.

## Background:

The anatomic proximity of the maxillary sinus to the posterior maxillary dentition establishes a close structural and functional relationship that facilitates direct communication between dental and sinonasal compartments [1]. The thin cortical bone of the sinus floor, frequent pneumatization into alveolar processes and root apices that occasionally protrude into the sinus cavity create potential pathways for microbial invasion and inflammatory spread [2]. Under physiological conditions, the mucociliary apparatus maintains sinus sterility, but disruption from dental disease, invasive procedures, or sinonasal obstruction compromises clearance and fosters infection [3, 4]. Odontogenic maxillary sinusitis often develops from apical periodontitis, periodontal disease, periapical cysts, or iatrogenic causes such as tooth extraction, implant placement and sinus augmentation [5]. In these scenarios, the microbial profile typically contains anaerobic oral commensals distinct from the flora of rhinogenic sinusitis, leading to treatment resistance if managed solely with conventional sinonasal regimens [6]. Clinically, odontogenic sinusitis may present with unilateral nasal obstruction, purulent discharge, malodor and dental tenderness, yet these features often overlap with rhinogenic disease, making diagnostic precision challenging [7]. Conversely, primary sinus pathology such as chronic rhinosinusitis-can cause secondary dental symptoms through contiguous inflammation, pressure-related bone changes, or neurogenic referral pain, sometimes mimicking odontogenic origin [8]. Prolonged sinonasal inflammation may also alter the alveolar bone environment, impacting dental implant stability and periodontal health. Shared risk factors such as smoking, diabetes and immunosuppression further complicate disease dynamics and hinder recovery [9]. Accurate differentiation relies on a multidisciplinary diagnostic approach, incorporating dental examination, otolaryngologic assessment and advanced imaging such as cone-beam computed tomography to delineate periapical lesions, sinus floor integrity and ostiomeatal complex patency [10]. Management requires addressing the primary source-definitive dental therapy for odontogenic cases or optimized sinonasal treatment for rhinogenic cases-while considering surgical intervention when conservative measures fail [11, 12]. Therefore, it is of interest to describe the interplay between sinus pathology and dental infections, highlighting the bidirectional disease mechanisms, diagnostic challenges and the importance of coordinated interdisciplinary care for optimal patient outcomes.

## Materials and Methods:

This retrospective observational study was conducted in a multidisciplinary setting involving otolaryngology and dental clinics of a tertiary care center. Clinical records from January 2018 to December 2023 were reviewed to identify patients diagnosed with either odontogenic maxillary sinusitis or sinus pathology with secondary dental involvement. Inclusion criteria comprised patients aged 18 years and older, confirmed diagnosis via clinical assessment and radiological imaging and complete treatment documentation. Exclusion criteria included patients with incomplete medical records, sinonasal tumors, facial trauma, or systemic conditions unrelated to infection that could confound outcomes. Data collected included demographic details, medical history (with emphasis on comorbidities s uch as diabetes and smoking), chief complaints, clinical examination findings, dental status and sinus involvement pattern. Imaging findings from cone-beam computed tomography (CBCT) or paranasal sinus CT scans were reviewed for each case, documenting the extent of sinus opacification, mucosal thickening, periapical lesions, oroantral communication and patency of the osteomeatal complex. Microbiological data, where available, were recorded to differentiate odontogenic from rhinogenic flora. Treatment details were retrieved from procedural records, encompassing dental interventions (endodontic therapy, extraction, implant removal), sinonasal procedures (functional endoscopic sinus surgery, antral lavage) and combined approaches. Follow-up records up to 12 months were examined for recurrence, resolution, or complications. For analysis, categorical variables such as sex distribution, laterality of disease and type of primary pathology were expressed as frequencies and percentages. Continuous variables such as age and duration of symptoms were presented as mean ± standard deviation. Comparative analysis between odontogenic and rhinogenic groups was performed using the chi-square test or Fisher's exact test for categorical variables and the independent t-test or Mann-Whitney U test for continuous variables, depending on normality of distribution. A p-value < 0.05 was considered statistically significant.

## Results:

The retrospective analysis included 186 patients, of whom 102 had odontogenic sinus pathology and 84 had rhinogenic origins. The age distribution was predominantly middle-aged adults, with a slight male predominance in both groups. Comorbidities such as diabetes mellitus and chronic periodontitis were significantly more prevalent in the odontogenic group. Clinical presentation patterns revealed that facial pain, nasal obstruction and purulent nasal discharge were common in both groups, but toothache and gingival swelling were significantly higher among odontogenic cases. Imaging findings on CBCT and CT scans demonstrated localized maxillary sinus opacification in odontogenic cases, whereas rhinogenic cases showed more diffuse involvement. Microbiological cultures indicated a higher prevalence of anaerobic bacteria in odontogenic cases and mixed aerobic-anaerobic growth in rhinogenic cases. Treatment modalities varied, with combined dental and ENT interventions being more frequent in odontogenic cases, while functional endoscopic sinus surgery (FESS) predominated in rhinogenic pathology. Follow-up data revealed a lower recurrence rate in cases receiving multidisciplinary management. The recurrence was more frequent in patients with poorly controlled diabetes and chronic periodontal disease. Overall, early diagnosis and targeted intervention improved clinical outcomes across both groups. Table 1 (see PDF) shows that middle-aged adults formed the largest group, with a slight male predominance and higher prevalence of diabetes mellitus in the odontogenic group. Table 2 (see PDF) demonstrates higher prevalence of toothache, gingival swelling and unilateral nasal obstruction in odontogenic sinus pathology compared to rhinogenic cases. Table 3 (see PDF) shows that odontogenic sinusitis cases predominantly had localized maxillary sinus opacification and adjacent dental pathology, while rhinogenic cases had diffuse sinus involvement. Table 4 (see PDF) reveals a higher prevalence of anaerobic bacterial isolates in odontogenic sinusitis, while rhinogenic cases more often showed mixed aerobic-anaerobic flora. Table 5 (see PDF) shows that odontogenic cases frequently required combined dental and ENT interventions, whereas FESS was more common in rhinogenic pathology. Table 6 (see PDF) demonstrates that odontogenic cases often presented later, with a higher proportion exceeding four weeks of symptoms. Table 7 (see PDF) reveals that recurrence and persistent symptoms were more frequent in odontogenic cases, particularly among patients with uncontrolled diabetes. Table 8 (see PDF) indicates that uncontrolled diabetes and chronic periodontitis were significantly associated with recurrence of symptoms. Table 9 (see PDF) shows that median time to resolution was longer in odontogenic cases, especially when treatment was delayed beyond two weeks. Table 10 (see PDF) highlights that multidisciplinary care yielded better symptom-free rates compared to single-specialty intervention.

## Discussion:

The present retrospective study examined the interrelationship between sinus pathology and dental infections, providing new insight into the overlapping clinical, microbiological and therapeutic aspects that shape disease outcomes. Findings from the cohort reinforce the established notion that odontogenic infections are an important but often under-recognized cause of maxillary sinus disease [13]. The demographic patterns observed, including a predominance of middle-aged adults and a slightly higher proportion of males, align with earlier epidemiological reports and underscore the need for targeted preventive and screening strategies in these populations [14]. Clinically, the differential between odontogenic and rhinogenic sinus disease was corroborated by symptom profile. Although the traditional sinonasal symptoms of nasal obstruction and discharge presented in both, odontogenic cases presented more commonly with toothache and gingival swelling, as a presentation of direct spread of infection from periapical or periodontal origin to the maxillary sinus [15]. Radiological evaluation supported the separations with regard to the odontogenic cases tending to be isolated maxillary disease next to the teeth affected and rhinogenic disease demonstrating more diffuse or multi-sinus patterns [16]. The microbiological picture provided an additional level of differentiation. Odontogenic sinusitis was dominated by more anaerobic organisms reflecting the anaerobic-dense flora of the oral cavity while rhinogenic cases exhibited more frequent mixed aerobic-anaerobic patterns [17]. There are direct therapeutic implications for these patterns since empirical antimicrobial selections should be directed against these microbiological predilections to aid efficacy and minimize the chances of persistence or recurrence. Management varied accordingly, with odontogenic cases being more likely to have combined dental treatment and ENT treatment or nasal sinus drainage such as extraction or endodontic treatment. Rhinogenic cases conversely, were more likely to have isolated functional endoscopic sinus surgery [18]. There was ample justification for multidisciplinary collaboration, in terms of long-term outcome improvements when dental and ENT care can be coordinated in the first instance. The research also provided prognostic factors. Uncontrolled diabetes mellitus, chronic periodontitis and smoking were consistently associated with a greater likelihood to experience recurrence, delayed recovery and poorer longer-term control [19]. This reinforces the importance of optimizing systemic disease and modifying risk factors as part of the management of sinusitis with an odontogenic cause. Also, late presentation was correlated with longer symptom duration, greater risk of developing chronicity or chronic sinusitis and delayed recovery, further supporting the timely diagnosis and commencement of treatment [20]. From a systemic perspective, this data lends credence to the concept of oral and sinus health as an interrelated construct. Ignoring odontogenic etiologies of chronic or recurrent sinusitis compromises treatment success and may subsequently lead to increased cost-to-healthcare, due to further interventions. Better multidisciplinary awareness, guideline-based referral processes and patient management and education may help to bridge this diagnostic and treatment chasm. This study highlights the need for a global, multidisciplinary approach to diagnosing and treating sinus disease, particularly in locations where dental infections are common and where systemic comorbidities are not uncommon. The addition of clinical, radiological, microbiological and example systemic risk factor characterization to clinical practice can potentially maximize treatment approaches and long-term patient outcomes.

## Conclusion:

A pathophysiologically plausible and clinically relevant relationship between sinus pathology and odontogenic infection through the early diagnosis of the etiological factors involved is relevant. Multidisciplinary, collaborative management, including dental, otolaryngological and systemic treatment, may maximize the potential for therapeutic success while limiting the risk of recurrence.
